# Subjective and psychological well-being of students of a University of the Third Age: Benefits of continuing education for psychological adjustment in the elderly

**DOI:** 10.1590/S1980-57642011DN05030010

**Published:** 2011

**Authors:** Tiago Nascimento Ordonez, Thaís Bento Lima-Silva, Meire Cachioni

**Affiliations:** 1 Bachelors in Gerontology from the University of São Paulo. Researcher at the Center for Study, Research and Extension in Gerontology of the University of São Paulo, São Paulo SP, Brazil; 2 Bachelors in Gerontology, Post-graduate in Neurosciences from the ABC School of Medicine, Santo André Foundation, Santo André SP, Brazil and Masters Student in Neurology at the University of São Paulo, School of Medicine, São Paulo SP, Brazil; 3 PhD in Education and MD, Specialist in Gerontology from the State University of Campinas, Campinas SP, Brazil. Professor on the Graduate course in Gerontology of the School of Arts, Sciences and Humanities of the University of São Paulo (EACH-USP), São Paulo SP, Brazil. Lecturer on the Post-Graduate Program in Gerontology of the School of Medical Sciences of the State University of Campinas, Campinas SP, Brazil. Chair of the Board for University Culture and Extension and Head of the University of the Third Age of the EACH-USP. Director of the Center for Study, Research and Extension in Gerontology of the University of São Paulo, São Paulo SP, Brazil

**Keywords:** continuing education, subjective well-being, psychological well-being, sense of psychological adjustment

## Abstract

**Objectives:**

The present study sought to characterize the degree of general satisfaction with life and degree of satisfaction on four domains: health, physical capacity, mental capacity and social involvement, and to determine the characteristics of self-reports of individuals enrolled on the program in relation to their psychological well-being focusing on the dimensions: autonomy, personal growth, control, positive relationships with others, purpose, personal acceptance and generativity, and to analyse the effect of time studying on level of well-being.

**Method:**

A total of 140 elderly students of a University for the Third Age took part in the study. The Global Satisfaction With Life Scale and the Self Development Scale (with six psychological well-being subscales) were applied. Continuous variables for the two groups were compared using the Mann-Whitney test. *Spearman’s* correlation coefficient was used to analyze the relationship among numeric variables. Internal consistency of the instrument scales was analysed by calculating *Cronbach’s alpha* coefficient.

**Results:**

Results showed that students who had attended the University of the Third Age for six months or longer had a higher level of satisfaction with life and greater psychological adjustment compared with new entrants to the same institution.

**Conclusion:**

The study results confirmed the positive effects of continuing education on the well-being of elderly and its contribution to successful aging.

## Abstract (pt)

**Objetivos:**

O presente estudo visou caracterizar o grau de satisfação geral com a vida e o grau de satisfação referenciada a quatro domínios: saúde, capacidade física, capacidade mental e envolvimento social; conhecer as características dos autorrelatos dos inscritos no programa em relação ao próprio bem-estar psicológico, focando as dimensões: autonomia, crescimento pessoal, domínio, relações positivas com os outros, propósito, autoaceitação e geratividade; analisar a influência do tempo no nível de bem-estar.

**Método:**

Participaram do estudo 140 idosos frequentadores de uma Universidade Aberta à Terceira Idade. Utilizou-se uma Escala de Satisf ação Geral Com a Vida e outra de Desenvolvimento Pessoal (com seis subescalas de bem-estar psicológico). Para comparação das variáveis contínuas entre os dois grupos foi utilizado o teste de Mann-Whitney. Para analisar a relação entre as variáveis numéricas foi utilizado o coeficiente de correlação de *Spearman*. Para avaliar a consistência interna dos instrumentos escalares foi calculado o coeficiente *alpha* de *Cronbach*.

**Resultados:**

Os dados revelaram que os participantes que frequentavam a Universidade Aberta à Terceira Idade há seis meses ou mais estavam altamente satisfeitos com a vida e com índices de ajustamento psicológicos superiores com relação aos que ingressaram atualmente na instituição estudada.

**Conclusão:**

Os resultados confirmaram os efeitos positivos da educação permanente para o bem-estar dos idosos e para um envelhecimento bem-sucedido.

Throughout the XX century, a worldwide increase in the elderly population gave rise to new ways of looking at old age. Influenced by technological and social progress which led to an increase in life expectancy and improved quality of life, the classic negative concept of longevity has been gradually revised.^1^ Concomitantly with this reconceptualization, the importance of life-long learning has gained relevance. In a report by Cachioni^1^ the author reiterates that learning is not an end in itself, but rather a means by which a person can fulfill a variety of personal goals and achieve growth throughout their life time.

Against this social background of change, methods of advanced research and opportunities for continued learning during old age, a new paradigm emerges of life span development, as proposed by Baltes, Reese and Lipsitt.^2^ This views aging as a phase which does not necessarily imply diseases and withdrawal, but instead regards old age as a phase of human development which allows not only the occurrence of losses but also gains, essentially of a compensatory nature. This paradigm places great emphasis on the role of education as an instrument for promoting successful aging, i.e. enjoying good quality of life biologically, psychologically and socially. Educational opportunities are indicated as important antecedents of evolutive gains in aging, for fostering greater social interaction, the sharing of experience and knowledge as well as promoting self-betterment.^3^

Based on the premise that intellectual, physical and social activities promote health, along with both psychological and social well-being in many countries, including Brazil, Universities are offering continued education programs aimed at mature and older adults at so-called Universities of the Third Age (U3A). In short, opportunities were created to enable people to live longer while enjoying quality of life. But what constitutes quality of life?

From a psychological perspective, Lawton^4^ introduced new concepts for quality of life. According to the author, quality of life has four dimensions (three of which are fundamentally psychological and subjective), namely: behavioral competence, environmental conditions, perceived quality of life and subjective well-being.

Quality of life is a set of appraisals and judgments which people make on certain domains of their lives. Some assessments are based on objective factors such as socioeconomic issues whereas others may be derived from subjective assessment, such in the case of subjective and psychological well-being. This conception justifies the introduction of subjective well-being as one of the most relevant elements in the study of quality of life.

According to Neri,^5^ quality of life “is one of the most studied terms in Gerontology and since the 1960s has been one of the main benchmarks in the field of aging”. The product of the assessment an individual makes on their capacities, environmental conditions and quality of life, based on personal criteria combined with the values and expectations which prevail in society. The most well-known indicator is satisfaction with life.

Assessment of this nature depends on the *self*, which corresponds to one’s self-knowledge and always comes into play on a temporal and comparative perspective. The ability to accommodate losses and to assimilate positive information about the *self*^5^ is pivotal to subjective well-being in old age.

Subjective well-being is a significant measure in this context since it is negatively associated with depression, fear of aging, loneliness, sense of loss of control, and feeling of being less competent or intact than other elderly people. Therefore, it can be considered an indicator of health, social integration and adjustment for the losses arising from aging, as reported by Neri.^5^

Psychological well-being, another concept of well-being, was also a focus of the present study. This arose as a result of criticisms of the fragility of the formulations underpinning subjective well-being and also as a response to psychological studies concentrating on unhappiness and suffering, yet overlooking the causes and consequences of positive functioning.^6^

Queroz^7^ reported that the interest in studying the characteristics and conditions inherent to good psychological adjustment in adult life and old age are not as generalized and valued as the interest in conditions causing unhappiness and suffering. Psychological well-being is an important indicator of psychological adjustment.

While subjective well-being is traditionally grounded in assessments of life satisfaction and in the balance between positive and negative affections (theoretical conceptions), the theories of psychological well-being are strongly rooted in psychological formulations centered on human development and structured on abilities to tackle the challenges of life.^6^

According to a summary published by Ryff,^8^ based on the analysis and review of the literature, the structure of an approach involving positive psychological functioning is based on several classic theories in psychology. Some are based on a clinical approach, including those that specifically address the phenomenon of individualization, self-realization, maturity and full functioning.

Ryff created a model of positive psychological functioning which represents this.^8^ The author points to the limitations of the literature regarding few studies examining the adjustment of adults and the elderly, evidenced by the absence of theoretical frameworks encompassing a larger number of dimensions of well-being and a certain negativism implicit in relation to the process of aging and old age, and to the scant attention dedicated to the resources and challenges of old age and to the possibilities of continued growth and development during this phase. Lastly, there is scant regard for the fact that conceptions of well-being are human constructs and therefore open to different competing definitions, as well as to cultural variation and historic change.

To define the construct of psychological well-being, Ryff^8^ developed an integrated model of positive psychological functioning which encompassed the factors of autonomy, positive relationships with others, personal acceptance, personal growth, purpose in life and mastering of the environment, which represents points of convergence of developmental theories (life-span), clinical theories on self-development as well as the theory of mental health. The author stresses that these three approaches provide the theoretical guidelines to understanding the construct of psychological well-being.^8^

Along the same lines, Ryff^8^ conducted qualitative studies involving adults and elderly individuals to ascertain their conception of happiness, a term the author adopted as synonymous with psychological well-being. Based on these reports, he proposed a multi-dimensional model of psychological well-being, also referred to as positive psychological functioning. According to the author, six factorial dimensions describe psychological well-being in terms of personal adjustment. These comprise positive relationships with others, autonomy, purpose in life, mastering the environment, personal growth and personal acceptance.

In short, the two concepts of subjective and psychological well-being, although similar, are different constructs. In their study of 3,032 Americans aged between 25 and 74 years, Keyes, Shmotkin and Ryff sought empirical evidence on for this relationship.^9^ Factorial analysis confirmed that the two concepts, although correlated, are different from one another. The results of this study revealed that an excellent state of well-being, defined by the authors are high subjective well-being and high psychological well-being, increased with age, schooling, strong dispositional traits such as extroversion and consciousness, but decreases with neuroticism, seen as a negative component of personality.^9^

Against this background, the primary aim of the present study was to characterize the degree of general satisfaction with life and degree of overall satisfaction in terms of the following four domains: health, physical and mental capacity, and social involvement. Secondly, the study sought to determine the characteristics of self-reports of individuals enrolled at the University of the Third Age of the School of Arts, Sciences and Humanities of the University of São Paulo (UnATI EACH USP), in relation to their psychological well-being with a focus on the dimensions: autonomy, personal growth, control, positive relationships with others, purpose, personal acceptance and generativity. Finally, the investigation analysed the influence of time studying at the University of the Third Age (U3A) on level of satisfaction with life and the sense of psychological adjustment.

## Methods

### Participants

The present study involved a sample comprising 140 students of the U3A of the School of Arts, Sciences and Humanities of the University of São Paulo corresponding to 95% of the total enrollers for the 1^st^ academic semester of 2009. The majority of the participants were women, aged between 60 and 69 years, had completed high school education, were retired and lived with the family (Table 1).

**Table 1:** Sociodemographic profile of participants.

Variable	Global			Groups					p-value
				**Group I**			**Group II**		
	**n**	**%**		**n**	**%**		**n**	**%**	
Gender									
Female	109	77.86		71	77.17		38	79.17	
Male	31	22.14		21	22.83		10	20.83	0.788^a^
Age (years)									
50 ≤ x ≤59	25	17.86		15	16.30		10	20.83	
60 ≤ x ≤ 69	89	63.57		63	68.48		26	54.17	
70 ≤ x ≤79	23	16.43		11	11.96		12	25.00	
x ≥80 years	3	2.14		3	3.26		0	0.00	
Mean (SD±)	64.57	(6.43)		64.38	(6.27)		64.94	(6.79)	0.248^b^
Schooling									
Primary school (unconcluded)	27	19.29		16	17.39		11	22.92	
Primary school (concluded)	27	19.29		19	20.65		8	16.67	
High school (unconcluded)	15	10.71		13	14.13		2	4.17	
High school (concluded)	41	29.29		25	27.17		16	33.33	
University (unconcluded)	12	8.57		6	6.52		6	12.50	
University (concluded)	18	12.86		13	14.13		5	10.42	
Mean (SD±)	8.98	(4.57)		9.08	(4.77)		8.79	(4.20)	0.967^b^
Marital status									
Single	14	10.00		10	10.87		4	8.33	
Married	69	49.29		44	47.83		25	52.08	
Separated	6	4.29		4	4.35		2	4.17	
Divorced	9	6.43		6	6.52		3	6.25	
Widow(er)	42	30.00		28	30.43		14	29.17	0.990^c^
Family income									
Up to 1 minimum wage	12	8.57		8	8.70		4	8.33	
From 1 to 2 min. wages	31	22.14		23	25.00		8	16.67	
From 2 to 3 min. wages	30	21.43		20	21.74		10	20.83	
From 3 to 4 min. wages	23	16.43		13	14.13		10	20.83	
From 4 to 5 min. wages	18	12.86		10	10.87		8	16.67	
From 5 to 10 min. wages	16	11.43		11	11.96		5	10.42	
More than 10 min. wages	10	7.14		7	7.61		3	6.25	0.465^b^
Active employment									
Yes	29	20.71		16	17.39		13	27.08	
No	111	79.29		76	82.61		35	72.92	0.179^a^
Retired or pensioner									
Yes	99	70.71		64	69.57		35	72.92	
No	41	29.29		28	30.43		13	27.08	0.679^a^
Living arrangements									
Alone	36	25.71		27	29.35		9	18.75	
With spouse only	29	20.71		17	18.48		12	25.00	
With child/children only	15	10.71		9	9.78		6	12.50	
With spouse and child/children	29	20.71		19	20.65		10	20.83	
With spouse. children and grandchildren	6	4.29		5	5.43		1	2.08	
Your children live with you	10	7.14		6	6.52		4	8.33	
Your children and grandchildren live with you	9	6.43		5	5.43		4	8.33	
Someone else lives with you	6	4.29		4	4.35		2	4.17	0.819^c^

a Chi-square test;

b Mann-Whitney test;

c Fisher's Exact test.

In order to determine benefits associated with time studying at the U3A, participants were divided into two groups: Group I – 92 recently enrolled students, having studied at the U3A for less than one academic semester; and Group II – 48 students that had studied for more than one academic semester. Mean age in Group I was 64.38 years (+ ±6.27) and in Group II was 64.94 years (+ ±6.79). Mean age for the overall sample of 140 participants was 64.57 years (+6.43). The minimum age found in the study sample was 50 years and the maximum 92 years (Table 1).

Regarding schooling, mean schooling at institutions of formal education was 9.08 years (±4.77) in Group I, and 8.79 (±4.20) years in Group II. Considering the total sample, mean schooling was 8.98 years (±4.57), with some individuals having studied for only one year and others for 23 years at institutions of formal education (Table 1).

The two groups (Groups I and II) were submitted to statistical tests to identify any differences in sociodemographics. The absence of differences between the two groups on all parameters, with the exception of study time at the U3A, confirmed the reliable comparison of the effect (negative or positive) of time studying at the U3A (Table 1).

### Procedures

Data was collected individually by previously trained graduate students of Gerontology using:

1. **Sociodemographic Questionnaire:** a questionnaire containing closed and mixed questions was used to gather sociodemographic data including gender, age, schooling, marital status and family income. The information for these variables was employed to check for possible influence on the study scales outlined below.
2. **Global Satisfaction With Life Scale (GSWLS) – Self-Anchoring Ladder:** This instrument, devised by Cantril in 1976, is an example of a single item scale. It is a graphically-based scale in the form of a ladder, with steps numbered from 1 to 10 representing a 10-point scale rising from worst life to best life.^10^
3. **Satisfaction with Life on Domains Scale (SLDS):** This consists of a scale comprising 12 items, each rated on a 5-point scale ranging from 1 (very dissatisfied) to 5 (very satisfied) created by Neri^11^for measuring subjective well-being indicated by satisfaction with life on four domains: health, physical capacity, mental capacity and social involvement.
4. **Self-Development Scale (SDS):** This comprises a scale adapted by Neri^12^ based on six subscales of psychological well-being produced by Ryff^13^ and three subscales of generativity,^14,15^ giving a 30-item scale. Eighteen items reflect the subscales of Ryff^13^ with three items for each of the six dimensions (positive relationship with others, personal growth, personal acceptance, autonomy, purpose in life, and control of environment) plus 12 items reflecting the concept of generativity, containing the subscales create, maintain and offer. Each item is assessed on a five-point scale (1-very slightly, 2-slightly, 3-moderately, 4-substantially and 5-very substantially).

For this study, the results of the SDS were analyzed according to the procedures outlined by Queroz,^7^ based on 5 factors (Factor 1: Self-realization, Personal Growth and Psychological Adjustment; Factor 2: Productivity; Factor 3: Care; Factor 4: Concern for future generations; Factor 5: Commitment to others). The author showed that 25 items is a sufficient number to measure psychological well-being. These are conceptually aligned with the theoretical definition of psychological well-being ^12^ and with the concept of generativity.^14,15^

### Investigation venue

The study was carried out at the School of Arts, Sciences and Humanities of the University of São Paulo in 2009. The School set up the University of the Third Age (U3A) in the second semester of 2006, making available the following activities to the elderly population. Complementary didactic-cultural activities – Gerontology Course: Workshops for the Promotion of Health and Quality of Life, Workshop in Health-related Care and Prevention of Diseases in the Third Age, Challenging Memory Workshop, Personal Growth Group; EACH Committee for Culture and University extension courses: East University of São Paulo Choir group. Regular subjects – Gerontology Course: Foundation in the Care Process in the Elderly; Leisure and Tourism course: Leisure and Tourism II Workshops.^16^

### Ethics aspects

The present study was approved by the Research Ethics Committee of the Institute of Psychology of the University of São Paulo under report no. 2008/020. All participants received a copy of the free and informed consent term, guaranteeing the right to voluntary participation and withdrawal from the study if and when they wished.

### Statistical analyses

In order to describe the sample profile according to the several study variables, frequency tables of categorical variables, and descriptive statistics including measures of position and dispersion of continuous variables, were built.

The Chi-square test was used to compare categorical variables between groups, and Fisher’s exact test was employed for expected values less than 5. Both tests compared the proportion observed of a given response with the proportion of responses obtained.

The *Kolmogorov-Smirnov* test was applied, the results of which confirmed that the continuous variables did not have a normal distribution (p-value<0.05), and therefore requiring the use of non-parametric tests. Thus, the Mann-Whitney test was used to compare continuous variables between the two groups. Spearman’s correlation coefficient was used to analyze the relationship among numeric variables. The internal consistency of the instrument scales was analysed by calculating Cronbach’s alpha coefficient. On Cronbach’s alpha test, values of 0.80 were taken to indicate high internal consistency and values between 0.60 and 0.79 as intermediate consistency. ^17^

The data were keyed into Version 3.1 of the Epidata Program and the Validate software was used to validate the data. Statistical analysis was carried out using the *Statistica* 7.0 (2004) software program. The level of significance adopted for the statistical tests was 5%, corresponding to a p-value<0.05

## Results

### Global satisfaction with life

Comparisons of Groups I and II for Global Satisfaction with Life yielded a mean score of 8.42 (±1.40) in Group II, exceeding the value obtained in Group I. However, this was not sufficiently significant to state that this group, comprising students who had been studying at the U3A longest, was more satisfied with life (Table 2).

**Table 2:** Comparison of scores on Global Satisfaction with Life Scale (GSWLS) between Group I and Group II.

Variable		Descriptive statistics						p-value
		**n**	**Mean**	**SD±**	**Minimum**	**Median**	**Maximum**	
Global satisfaction with life	Group I	92	8.07	1.36	5.00	8.00	10.00	0.146
	Group II	48	8.42	1.40	4.00	8.00	10.00	
	Total	140	8.19	1.38	4.00	8.00	10.00	

### Satisfaction with life on domains

The results of the analysis of the internal consistency of the Satisfaction with Life on Domains Scale (SLDS) are presented first. Cronbach’s alpha coefficients were used as an indicator of reliability of the SLDS for the whole sample (n=140), Group I (n=92) and Group II (48). Table 3 shows the total coefficient for the SLDS and for each group.

**Table 3:** Cronbach's coefficients for Satisfaction with Life on Domains Scale (SLDS).

Domains	N°. of items	Coefficients of total sample	Coefficients of Group I	Coefficients of Group II
Health	3	0.773	0.766	0.759
Physical capacity	3	0.809	0.780	0.851
Mental capacity	3	0.847	0.831	0.858
Social involvement	3	0.874	0.830	0.918
Total	12	0.882	0.867	0.872

The indices given showed that the SLDS had high consistency since alpha values greater than 0.70 indicate high structural coherence.^17^ Comparison of scores on the SLDS and time spent studying at the U3A found that students who had undertaken activities for longer were more satisfied than recent entrants (Table 4). The difference between the groups in terms of social involvement was noteworthy (p-value=<0.001).

**Table 4:** Comparison of scores on Satisfaction with Life on Domains Scale (SLDS) between Group I and Group II.

Variable		Descriptive statistics						p-value
		**n**	**Mean**	**SD±**	**Minimum**	**Median**	**Maximum**	
Health	Group I Group II Total	92 48 140	3.67 4.02 3.79	0.77 0.66 0.75	1.33 2.67 1.33	3.67 4.00 4.00	5.00 5.00 5.00	0.010
Physical capacity	Group I Group II Total	92 48 140	3.64 3.92 3.73	0.75 0.73 0.75	2.00 1.33 1.33	3.67 4.00 3.67	5.00 5.00 5.00	0.045
Mental capacity	Group I Group II Total	92 48 140	3.58 3.98 3.72	0.81 0.71 0.80	1.00 2.00 1.00	3.67 4.00 3.67	5.00 5.00 5.00	0.004
Social involvement	Group I Group II Total	92 48 140	3.72 4.20 3.89	0.80 0.90 0.86	2.00 1.00 1.00	4.00 4.33 4.00	5.00 5.00 5.00	<0.001
SLDS / Total	Group I Group II Total	92 48 140	3.65 4.03 3.78	0.58 0.55 0.60	1.92 2.33 1.92	3.74 4.17 3.87	5.00 5.00 5.00	<0.001

### Sense of psychological adjustment

Similarly, Cronbach’s alpha coefficient was used to measure the internal consistency of the Self-Development Scale - SDS and verify the internal reliability of responses on the instrument. Alpha values greater than 0.70 indicate high consistency.^17^ Table 5 depicts the Cronbach’s alpha coefficients of the SDS for the whole sample (n=140) and the two student groups: recently-enrolled students (n=92) and veteran students (n=48).

**Table 5:** Cronbach's coefficients for Self-Development Scale - SDS

Scale / Domains	N°. of items	Coefficients of total sample	Coefficients of Group I	Coefficients of Group II
SDS / Factor 1	12	0.832	0.797	0.865
SDS / Factor 2	4	0.752	0.735	0.788
SDS / Factor 3	3	0.574	0.545	0.609
SDS / Factor 4	3	0.549	0.506	0.623
SDS / Factor 5	3	0.361	0.388	0.316
SDS / Total	25	0.833	0.820	0.835

Factor 1: Self-realization, Personal Growth and Psychological Adjustment; Factor 2: Productivity ;Factor 3: Care; Factor 4: Concern for future generations; Factor 5: Commitment to others.

The indices showed that scale items achieved high internal consistency for Factors 1, 2 and total SDS. Factors 3, 4 and 5 however, failed to attain the coefficient of 0.70, indicating insufficient internal consistency to be considered part of the same concept of construct.^7^

Comparison of Groups I and II revealed statistically significant differences (Table 6), where subjects participating in U3A activities for more than one academic semester scored higher on Factor 1 – Self-realization, Personal Growth and Psychological Adjustment (p-value=0.001) and on total SDS (p-value=0.016).

**Table 6:** Comparison of scores on Self-Development Scale - SDS between Group I and Group II.

Variable		Descriptive statistics						p-value
		**n**	**Mean**	**SD±**	**Minimum**	**Median**	**Maximum**	
Factor 1	Group I Group II Total	92 48 140	4.08 4.34 4.17	0.47 0.49 0.49	2.67 2.83 2.67	4.08 4.38 4.17	5.00 5.00 5.00	0.001
Factor 2	Group I Group II Total	92 48 140	3.36 3.38 3.37	0.87 0.90 0.88	1.00 1.00 1.00	3.25 3.50 3.50	4.75 5.00 5.00	0.800
Factor 3	Group I Group II Total	92 48 140	3.83 4.02 3.90	0.66 0.65 0.66	2.00 1.67 1.67	4.00 4.00 4.00	5.00 5.00 5.00	0.073
Factor 4	Group I Group II Total	92 48 140	3.95 4.04 3.98	0.84 0.91 0.86	1.67 2.00 1.67	4.00 4.00 4.00	5.00 5.00 5.00	0.406
Factor 5	Group I Group II Total	92 48 140	4.18 4.21 4.19	0.64 0.60 0.62	2.33 2.33 2.33	4.33 4.33 4.33	5.00 5.00 5.00	0.894
SDS/Total	Group I Group II Total	92 48 140	3.93 4.10 3.99	0.41 0.43 0.43	2.68 2.71 2.68	3.96 4.18 4.08	4.71 5.00 5.00	0.016

Note: Factor 1: Self-realization, Personal Growth and Psychological Adjustment; Factor 2: Productivity; Factor 3: Care; Factor 4: Concern for future generations; Factor 5: Commitment to others.

### Correlation of scale scores and sociodemographic variables

Finally, rank correlations were calculated (Spearman’s test) for the distribution of responses on the scales and the sociodemographic variables of age, schooling and family income (Table 7).

**Table 7:** Correlation between scale scores and sociodemographic variables: age, schooling and income.

Variable	n	Sociodemographic variables							
		**Age**			**Schooling**			**Family income**	
		**r**	**p-value**		**r**	**p-value**		**r**	**p-value**
Global Satisfaction with Life Scale	140	0.19	0.028		-0.13	0.130		-0.01	0.908
SLDS / Health	140	0.09	0.271		0.14	0.101		0.15	0.067
SLDS / Physical capacity	140	0.09	0.315		0.13	0.129		0.16	0.062
SLDS / Mental capacity	140	-0.11	0.190		0.32	0.000		0.21	0.014
SLDS / Social involvement	140	0.02	0.784		-0.02	0.854		-0.05	0.546
SLDS / Total	140	0.01	0.884		0.17	0.041		0.15	0.087
SDS / Factor 1	140	0.10	0.258		0.10	0.234		-0.03	0.757
SDS / Factor 2	140	0.05	0.580		-0.10	0.228		-0.07	0.435
SDS / Factor 3	140	0.04	0.673		0.05	0.548		0.01	0.945
SDS / Factor 4	140	-0.15	0.080		0.30	0.000		0.18	0.029
SDS / Factor 5	140	0.16	0.064		0.06	0.446		-0.06	0.460
SDS / Total	140	0.05	0.582		0.11	0.192		-0.01	0.863

r: Spearman's correlation coefficient; n: number of individuals.

Age was found to correlate positively with scores on the Global Satisfaction With Life Scale - GSWLS (p-value=0.028), where the higher the schooling and income, the better the sense of mental capacity (p-value<0.001and p-value=0.014). Schooling also showed a positive correlation with total score on the Satisfaction with Life on Domains Scale – SLDS (p-value=0.041). The score for Factor 4 on the SDS (Concern with future generations) correlated with both schooling and income (p-value<0.001 and p-value=0.029).

## Discussion

With regard to the overall sample, results showed that global satisfaction with life was positively correlated with age. Satisfaction was found to be greater among older elderly than younger elderly. This finding is consistent with results in the literature from studies on adults and elderly that disclose a tendency for increased levels of satisfaction in comparisons of younger adults with older adults and elderly.^7,10,18,19^ This phenomenon can be explained by the fact that younger generations are always more demanding than proceeding ones. The elderly, besides being less demanding, tend to be better at matching, through an adaptive process, their goals to the resources and competencies available to them.

Another important general factor concerns schooling and income of participants. In this study, individuals with higher educational level were shown to assess their lives more positively. Corroborating these findings, Joia, Ruiz and Donalisio^20^, in an interview of 365 elderly from the municipality of Botucatu , São Paulo and employing proportional and random stratified sampling to describe the factors associated to degree of satisfaction with life among the elderly population, demonstrated this same relationship, concluding that educational level was strongly associated with degree of satisfaction with life.

The present study also found income to be positively correlated with Factor 4 (Concern with future generations) on the Self-Development Scale. According to Queroz,^7^ Factor 4 shows concern with future generations and manifesting by the wish to pass on something of themselves to the current and next generations, being productive and considerate in relation to them. McAdams, Hart & Maruna^21^ affirmed that the generativity concern was positively associated with measures of psychological well-being and with personality characteristics – extroversion, affability, openness to experiences, emotional stability, and low tendency for neurosis.

Comparing scores for total SDS and Factor 1 – self-realization, personal growth and psychological adjustment, the participants that had attended the U3A for more than six months had higher indices of personal development, self-realization, personal growth and psychological adjustment than those participating for a shorter period. For Factor 1 of the SDS, individuals tend to describe their personality as having continuity with integration and personal growth terms as well as openness to new experiences, realization of their own potential and pursuit of the goal of personal excellence.^22^

Although no causal relationship can be firmly established, U3A study time of over six months proved a good predictor of good level of subjective and psychological well-being. Concordant with the results of the present study, previous investigations have reported the positive effect of taking part in education programs conferred to the elderly, in terms of improvement in their physical and mental health, as well as their attitudes and social relationships.^1,23-30^

Cachioni and Neri^29^ highlighted the transforming characteristics of education for the elderly, favoring successful aging by promoting cognitive flexibility, personal adjustment, subjective well-being and social image of this group. The authors drew on data from Brazilian research on education initiatives among elderly currently being conducted in Brazil, and to call attention to the need to train specialized professionals to cater for this clientele.

The study sought to determine the characteristics of self-reports of individuals enrolled at the University of the Third Age of the School of Arts, Sciences and Humanities of the University of São Paulo (UnATI EACH USP), in relation to their psychological well-being with a focus on the dimensions: autonomy, personal growth, control, positive relationships with others, purpose, personal acceptance and generativity. Finally, the influence of time studying at the U3A on level of satisfaction with life and sense of psychological adjustment was analyzed.

Based on the results of this study, it can be concluded that the participants of the University of the Third Age of the School of Arts, Sciences and Humanities of the University of São Paulo benefited from the program and enjoyed successful aging, mirroring the results found by similar studies on the theme.^1,22-26^

Analysis of time studying at the U3A revealed that the students who had been longer on the program run by the institute studied, exhibited higher levels of subjective and psychological well-being. These positive indices show that attracting this group and offering compelling subjects (knowledge builders, personal development, increased social contact, learning to be able to assist others, and occupy free time) results in them staying on as students, where the satisfaction and benefits gained extend into other areas of life.

Future studies on the theme could provide continuation of these investigations and explore other variables such as cognition, beliefs held about old age, and quality of life, while also centering on the potential benefits of life-long learning.
